# Biohybrid cochlear implants: neural interfaces, regenerative pathways, and translational benchmarks

**DOI:** 10.1186/s12984-026-02069-5

**Published:** 2026-06-30

**Authors:** Akihiro J. Matsuoka, Andrew N. Carpino, Audrey Meador, Beatriz O. Nicolau, Gabriela M. Fortuño, Huimin Zhu, Tifany T. Nguyen, Kiersten R. Russ, Hudson Liu, Jae Joon Kim, Winston Loh, James Friend

**Affiliations:** 1 Department of Otolaryngology-Head and Neck Surgery, University of California San Diego, 9444 Medical Center, MC7895, La Jolla, 92037 CA USA; 2 Department of Bioengineering, University of California San Diego, 9500 Gilman Dr, San Diego, 92093 CA USA; 3 Department of Bioengineering, University of California Los Angeles, 20 Westwood Plaza, Los Angeles, 90095 CA USA; 4 School of Medicine, Georgetown University, 3900 Reservoir Rd NW, Washington DC, 20007 DC USA; 5 Department of Mechanical Engineering and Materials Science, Washington University in St. Louis, 1 Brookings Drive, MSC 1185-208-125, St. Louis, 63130 MO USA

**Keywords:** Cochlear implant, Human induced pluripotent stem cells, Biomaterial, BDNF

## Abstract

Cochlear implants (CIs) restore hearing by electrically stimulating the auditory nerve, yet the millimeter-scale electrode-neuron separation limits spatial selectivity and temporal fidelity. Rather than relocating the electrode array, we propose a biohybrid strategy that preserves the conventional scala tympani insertion and closes this gap biologically by interposing living neural constructs between the array and the modiolus. This approach leverages the Canaliculi Perforantes of Schuknecht (CPS) to deliver spatially confined biochemical, mechanical, and electrical cues from the scala tympani, biasing spiral ganglion neurites toward contacts while respecting surgical practicality. We review CPS microanatomy and transport, auditory neuron/stem cell-regeneration prospects, hydrogel/assembloid niches, surface and material stacks, and conclude with translational benchmarks, constraints, and a near-mid-long-term roadmap.

**Supplementary Information:**

The online version contains supplementary material available at https://doi.org/10.1186/s12984-026-02069-5.

## Background

### Introduction

Cochlear implants (CIs) are among the most successful neural prostheses in clinical use. Still, their plateau in performance points to a central limitation of contemporary design: the submillimeter-millimeter-scale distance between stimulating contacts in the scala tympani (ST) and the excitable elements of the spiral ganglion neurons (SGNs) within Rosenthal’s canal [1–3]. Even with perimodiolar arrays, this separation attenuates voltage gradients at target neurons and broadens current spread between neighboring contacts. The consequence is reduced channel selectivity—the ability of an individual electrode to activate a spatially restricted SGN population without coactivating neurons assigned to neighboring frequency channels, and therefore the number of effective (perceptually independent) frequency channels available to the listener [4–6]. Loss of channel selectivity, together with degraded temporal coding, limits the fidelity of speech-in-noise understanding, music perception, and spatial hearing [1, 2, 7]. In short, the field faces an engineering interface problem at miniature anatomical scales [8].

**Fig. 1:**
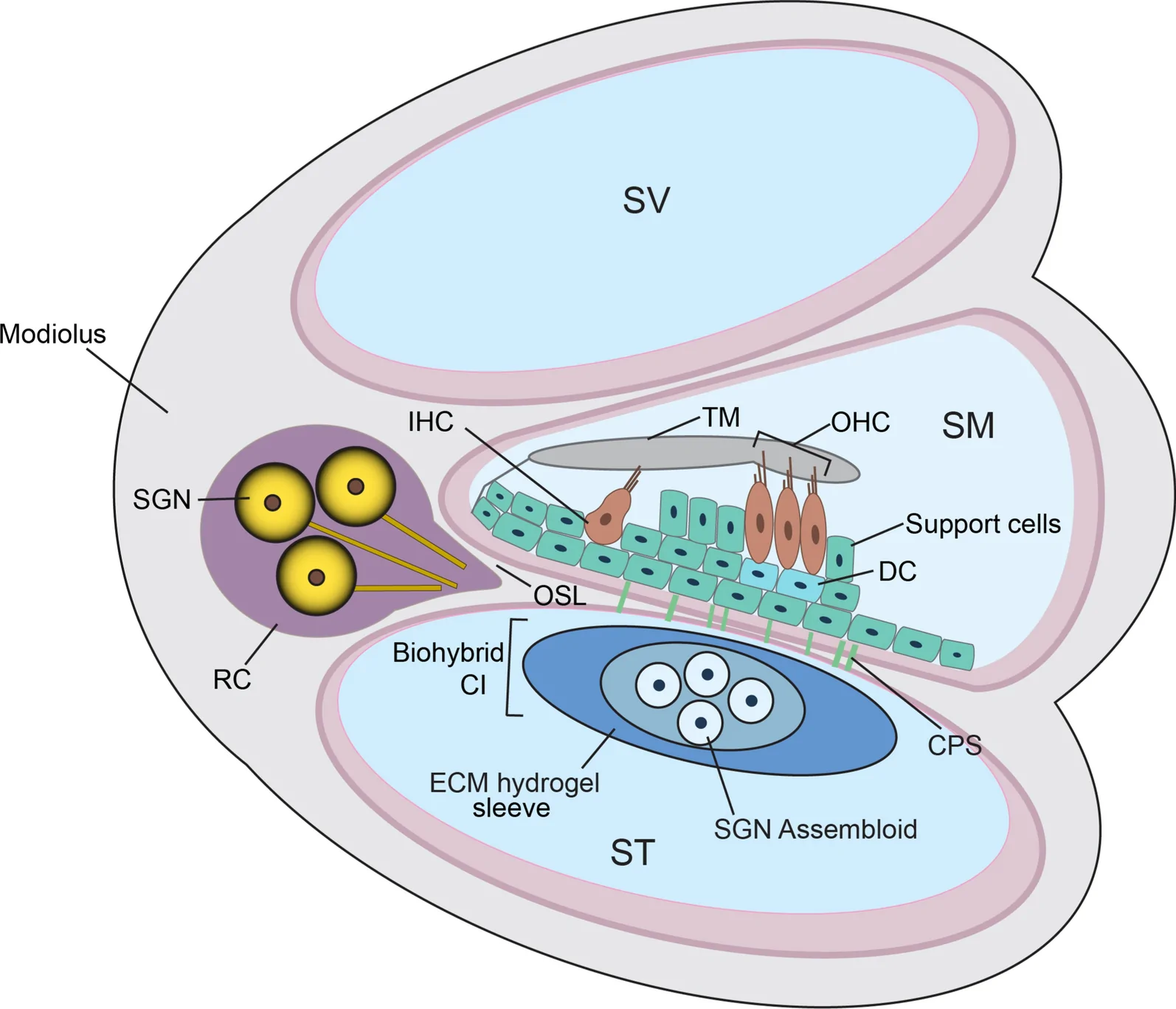
Cochlear micro-anatomy and a 2D concept of a biohybrid cochlear implant (CI). Basal-turn cross-section depicting the three scalae–scala vestibuli (SV), scala media (SM), and scala tympani (ST). The organ of Corti is shown within the SM and contains inner hair cells (IHC) and outer hair cells (OHC), with Deiters’ cells (DC) and other support cells beneath; the tectorial membrane (TM) overlies the hair-cell stereocilia. Spiral ganglion neurons (SGNs) reside in Rosenthal’s canal (RC) within the modiolus, with peripheral processes coursing through the osseous spiral lamina (OSL) toward the organ of Corti. The native canaliculi perforantes of Schuknecht (CPS, shown as green vertical channels piercing the OSL) provide a parallel set of micro-conduits between the ST and the SGN region. The proposed biohybrid CI lies in the ST and is co-implanted with a thin, position-stable extracellular matrix (ECM) hydrogel sleeve that hosts small SGN-based assembloids (3D multicellular constructs of hiPSC-derived otic neurons and supporting cells; see Sect. "Human stem-cell derived assembloids and hydrogel scaffolds for a living cochlear implant"); the sleeve is positioned adjacent to, but does not coat or cover, the electrical contact faces. The device is aligned to the CPS field along the medial ST wall, where the CPS are envisioned as native micro-conduits targeted with localized biochemical and electrical cues to encourage neurite ingress toward the modiolus and thereby narrow the electrode-neuron gap biologically rather than geometrically. The schematic is a conceptual layout showing the spatial relationships among the cochlear scalae, the OSL/CPS field, the SGN cell bodies in Rosenthal’s canal, and the proposed biohybrid CI components in the ST. Cellular details (hair-cell stereocilia, SGN peripheral processes) are drawn in their reference (intact) state for orientation; the deafened, partially-degenerated state characteristic of CI candidates and the resulting electrode-neuron gap that the biohybrid strategy seeks to bridge biologically are discussed in Sects. "Background"–"Anatomical rationale". Schematic is stylized and not to scale

A promising yet under-recognized anatomical substrate is the network of modiolar microchannels historically described as the Canaliculi Perforantes of Schuknecht (CPS) [9]. Contemporary CI electrode arrays are inserted into the ST through either a cochleostomy or, more commonly, the round-window membrane, and advanced along the lateral or medial (modiolar) wall for approximately 20–30 mm [10–12]. Even the most aggressive perimodiolar designs, which hug the modiolar wall, remain separated from SGN cell bodies in Rosenthal’s canal by the intervening osseous spiral lamina (OSL) and perilymph–typically 0.5–2 mm [10, 13]. Strategies that attempt to further reduce this distance through deeper modiolar drilling risk damaging SGN peripheral processes and compromising residual hearing [7, 14]. Traversing the OSL bone without drilling, however, is already anatomically feasible: the lamina is pierced by a dense network of microchannels—the CPS—that connect ST perilymph to the SGN-containing modiolus [15, 16]. These channels are too small (0.2–27 *μ *m) to accept an electrode, but they are precisely sized to serve as conduits for neurotrophins, construct-derived trophic factors, and regenerating neurites [17, 18]. Our working hypothesis is therefore not to move the electrode closer, but to bridge the gap biologically: exploit the CPS as native micro-conduits, paired with tuned chemical, mechanical, and electrical cues, to bring addressable neural elements to the existing electrode (Fig. 1) [19, 20].

The notion of a “living” or biohybrid implant aligns with broader trends in regenerative bioelectronics, wherein devices co-integrate with host or grafted tissue to recover function and enable closed-loop control [21–23]. Recent perspectives argue that effective interfaces deliver three classes of cues: (i) chemical, as spatiotemporally controlled trophic factors, genes, or vesicles; (ii) mechanical, such as stiffness, roughness, and topography to control cell behavior and neurite trajectories; and (iii) electrical, in the form of fields and stimulation paradigms to modulate migration, growth, and maturation [22, 24]. The cochlea is a stringent test ground for these ideas because it demands long-term stability in a compact, fluid-filled, delicate environment with strict constraints on insertion mechanics, biocompatibility, and serviceability [7].

### Impact of auditory deprivation on CI outcomes

In addition to the electrode-neuron interface limits, outcomes with conventional CIs decline as the duration of deafness increases. In post-lingually deaf adults, meta-analytic evidence shows a consistent negative association between years of auditory deprivation and postoperative open-set speech recognition [25]. In congenital or early-onset deafness, delayed implantation beyond the early sensitive period of auditory cortical development yields poorer speech and language outcomes on average, with many late-implanted pre-lingual users attaining only limited open-set recognition despite gains in audibility and quality of life [26]. Mechanistically, prolonged deprivation is associated with progressive SGN loss in humans and animals and central reorganization that reduces neural responsiveness to electrical input; moreover, the efficacy of neurotrophin-based rescue diminishes with longer deafness duration [14, 27]. These observations motivate biohybrid CI strategies that pair electrical stimulation with targeted biological support to preserve or restore the electrode-neuron interface discussed in this review.

### Cochlear implants and their limitations

CIs bypass defective outer and inner hair cells and directly stimulate SGNs [1, 2]. Nevertheless, many CI users struggle with speech in noise and with percepts that rely on fine temporal and spectral cues, such as prosody or tone quality [28, 29]. The combination of residual electrode-neuron separation, ongoing SGN decline (see "Impact of auditory deprivation on CI outcomes" section), and limited preoperative diagnostics that distinguish predominant neurite versus hair-cell pathology complicates patient-specific interface optimization [7]. Despite the range of preclinical strategies reviewed in the following subsection, none has yet matured into a safe, scalable, clinically validated method to reliably narrow the electrode-neuron gap or improve perceptual outcomes.

### Current strategies for enhancing CI performance

To address these challenges and optimize electrode-neuron interactions, several approaches have been explored: localized neurotrophin delivery (e.g., neurotrophin via viral vectors, small-molecule analogs, or encapsulated cells), stem cell-based therapies, and advanced biomaterials and coatings designed to lower impedance, add compliance, and present bioactive cues [7, 30–34]. While encouraging, most molecular strategies rely on passive diffusion through cochlear fluids, limiting sustained spatial precision at the modiolus. The biohybrid framework introduced in "Introduction" section is designed to overcome this limitation by pairing living neural constructs with CPS-targeted delivery, as motivated by the epidemiological and anatomical context developed in the following sections.

### Overview

This review advances a biohybrid strategy to address the electrode-neuron interface, prioritizing co-integration with living tissue rather than solely pushing electrodes closer or adopting more aggressive insertion paths. Section "Epidemiology and economic burden of hearing loss" summarizes the epidemiology and economic burden of hearing loss to motivate higher-fidelity interfaces. Section "Anatomical rationale" defines the modiolar microanatomy relevant to electrode–neuron coupling and the routes that matter for neurite growth. Section "Selecting the bridge neuron: which cells belong in the CI–SGN gap?" considers which neuronal phenotype should occupy the modiolar space and argues for an SGN-lineage bridge cell. Section "Human stem-cell derived assembloids and hydrogel scaffolds for a living cochlear implant" reviews 3D culture formats—spheroids, organoids, and SGN assembloids—and the hydrogel scaffolds that host them. Section "Biomaterial interfaces for next-generation cochlear implants" surveys electrode materials and device architectures relevant to a biohybrid array. Section "Surface modifications to enhance neuron-to-electrode interactions" outlines surface treatments and biochemical modifications that enable the use of hiPSC-derived cells with electrode arrays. Section "Conclusions and translational outlook" proposes translational benchmarks and constraints, including surgical feasibility and regulatory considerations for combination products, a near-term experimental roadmap, and open questions.

By consolidating anatomical clarity with emerging interface strategies, our goal is to provide a practical framework for researchers developing biohybrid approaches to cochlear implantation, and a shared language for neurotology, neuroengineering, and materials communities working toward this common objective.

## Epidemiology and economic burden of hearing loss

Hearing loss is a major public health concern, affecting 13–15% of Americans to some degree. Approximately one in eight individuals aged 12 or older (13%, about 30 million) has measurable hearing loss in both ears, and 15% of U.S. adults (37.5 million) report at least some trouble hearing [2, 35–37]. These figures surpass the prevalence of several other common health conditions, such as diabetes and cancer, underscoring the substantial impact of hearing impairment on public health resources and quality of life [38].

Within this broader population, cochlear implantation is indicated only for a specific audiological subgroup. Current FDA and CMS criteria define adult CI candidacy as bilateral moderate-to-profound sensorineural hearing loss with limited benefit from appropriately fit hearing aids; in contemporary clinical practice, this is operationalized by AzBio sentence recognition scores of *≤ *60% in the best-aided condition and *≤ *50% in the ear to be implanted [39]. An estimated 2.2 million U.S. adults aged 20 or older meet audiometric criteria for severe-to-profound SNHL [40], yet penetration among candidates remains low—most published estimates fall between 2 and 13%, meaning the majority of adults who could benefit from a CI never receive one [41].

Beyond the societal costs of hearing loss, which include reduced productivity and additional learning resources required to teach young people with hearing loss [42], there are significant direct and indirect costs associated with hearing loss. Direct costs include medical consultations; hearing aids at $1000–$4000 per pair; cochlear implants, $30,000–$100,000 per patient; audiological services; speech-language therapy; and hearing device maintenance. Indirect costs are $176 billion annually due to lost productivity: adults with hearing loss have 1.98-fold higher odds of unemployment and annual income losses of up to $30,000 per person. These costs impose a substantial burden at the individual and population levels [38, 42–46]. More recent longitudinal analysis shows that hearing loss is associated with lower earnings growth among working-age U.S. adults [47].

[48] found the global annual economic cost of hearing loss exceeded $981B in 2019, with 47% of these costs associated with quality-of-life losses and 57% incurred outside high-income countries [48, 49]. The World Health Organization estimates that increasing ear and hearing care interventions to 90% coverage over the next ten years requires an additional investment of $238.8B, an expensive effort that would nonetheless provide over $2T in productivity gains by 2030 [49]. From the individual patient perspective, lifetime healthcare costs for someone born with severe to profound hearing loss are estimated at $489K, but providing a cochlear implant before 18 months of age reduces this to $391K (95 % CI $312K–$471K), yielding a net lifetime savings of $98K [50].

In 2023, the global cochlear implant market was valued at $2.19B and is expected to grow to $8.06B by 2032. This surge of value reflects an annual growth rate of 15.6% expected from 2024 to 2032 [51, 52]. This increase in hearing loss is driven by factors such as genetics, noise exposure, and aging, where 25% of adults 60 years of age and older are affected by disabling hearing loss, and over one billion young adults are at risk of permanent hearing loss [38, 53]. Cochlear implants offer many benefits to hearing restoration, but there is still a high barrier, as a cochlear implant procedure can cost $50K to $100K.

In the US, device acquisition alone typically ranges $34–$38K before facility, professional, and rehabilitation fees, and total episode costs can be substantially higher; in Australia, median total treatment costs were around AUD$34.8K across centers [54, 55]. Key companies within the cochlear implant field include Cochlear Limited (Australia), Advanced Bionics (Sonova Holding AG, Switzerland), Zhejiang Nurotron Biotechnology Co., Ltd. (Hangzhou, China), and MED-EL Medical Electronics (Innsbruck, Austria) [56]. There are high barriers to entry into the US market due to FDA regulations regarding the use of novel long-term implants as class III devices, and the associated high research and development costs [57]. Nonetheless, given the market size, opportunities for new cochlear implants exist as innovative technologies are created. Critically, the rationale for pursuing biohybrid CI strategies is functional, not economic. A biohybrid device will initially be at least as costly as a conventional CI, but it targets the electrode-neuron interface limitations—channel selectivity, speech-in-noise understanding, music perception, and spatial hearing—that constrain CI benefit for every user regardless of cost (see "Cochlear implants and their limitations"). In summary, these challenges support the urgent need for next-generation cochlear implants, which the following sections will address from an anatomical and technological perspective.

## Anatomical rationale

### Cochlear scalae in brief

**Fig. 2:**
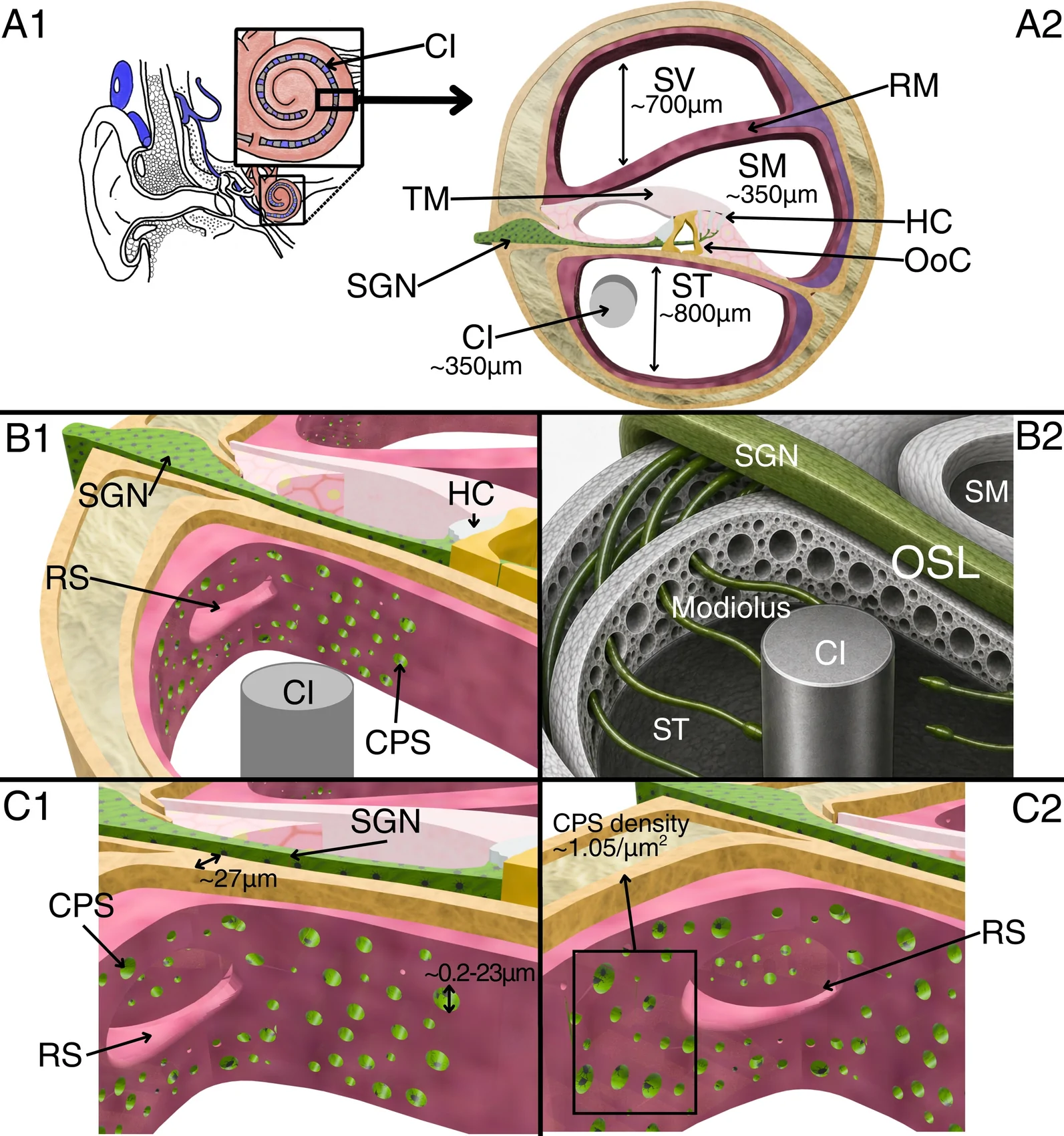
3D-Cochlear micro-anatomy and Canaliculi Perforantes of Schuknecht (CPS) in relation to a cochlear implant (CI). **A1** Whole-cochlea overview indicating the location of the basal-turn cross-section shown in** A2**; the boxed inset and arrow indicate the cross-sectional viewing direction. **A2** Basal-turn cross-section showing the three scalae–scala vestibuli (SV), scala media (SM) bounded by Reissner’s membrane (RM), and scala tympani (ST)—with the organ of Corti (OoC) containing inner and outer hair cells, Hensen’s cells (HC), and the tectorial membrane (TM). Indicated lumen heights ($\approx {700}{\,\mu \text {m}}$ SV, $\approx {350}{\,\mu \text {m}}$ SM, $\approx {800}{\,\mu \text {m}}$ ST) are mid-section values for illustrative purposes; published full vertical dimensions of the basal ST range up to *≈ 1.2*–1.4 mm [58, 60]. The CI ($\approx {350}{\,\mu \text {m}}$ in cross-section) is rendered at a representative ST position; in clinical practice CI placement varies between modiolus-hugging (perimodiolar) and lateral-wall trajectories with corresponding electrode-to-SGN distances ranging from *≈ 0.5*–1 to *≈ 1.5*–2 mm (see "Anatomical rationale"). The osseous spiral lamina (OSL) and CPS field separate the ST from the spiral ganglion neurons (SGNs) housed within Rosenthal’s canal of the modiolus. **B1** Oblique transverse cut-away illustrating the field of CPS (green pores in the modiolar OSL bone) along the retrocolumnar space (RS) between ST and the SGN region; the CI is shown as a stylized cylinder in the ST. **B2** Complementary longitudinal cut-away of the same modiolar region; the SGN bundle and its peripheral processes are rendered in green, visually distinct from the textured cream/beige OSL bone, to clarify that the SGNs are a discrete neural structure traversing—not continuous with—the bony lamina. The longitudinal view emphasizes that native SGN peripheral processes course through the modiolus and pierce the OSL via the CPS, motivating the bridging concept developed in "Anatomical rationale"–"Selecting the bridge neuron: which cells belong in the CI–SGN gap?"; the CI in** B2** is rendered at the same stylized scale used in** B1**. **C1, C2** Higher-magnification views of the CPS field; individual CPS openings span $\approx {0.2}{27}{\upmu }$ in diameter, the SGN layer thickness at this level is $\approx {27}{\,\mu \text {m}}$, and local areal density is $\approx 1.05 \pm 1.03$ pores per ${1000}{\mu \textrm{m}^{2}}$ in the basal turn (the panel-**C2** label “CPS density $\sim 1.05/\mu \textrm{m}^2$” refers to this same per-1000-µm^2^ value). Citations: [9, 17, 18, 61–63]. Figure** B2** was prepared in SolidWorks, Blender, and Canva from human-drawn source images; Google Gemini was used in a non-generative capacity to enhance image sharpness and contrast. No content was generated by AI

Within the cochlea of the bony labyrinth, the perilymph-filled scala vestibuli (SV) and ST spiral around the modiolus and are continuous at the helicotrema; between them lies the endolymph-filled scala media (SM) (i.e., the cochlear duct) of the membranous labyrinth (Fig. 2A1, A2). Conventional cochlear implants occupy the ST (Fig. 2A1), placing their contacts within millimeters—and rarely microns—of SGNs embedded in Rosenthal’s canal (dendrite) and modiolus (cell body) (Fig. 2 A2) [15, 58]. Importantly, the typical electrode-to-SGN distances are around 0.51mm for modiolus-hugging arrays and up to $\approx {1.5}{2}\text {mm}$ for lateral wall placements [10]. Even in the best-case scenario, a gap on the order of $10^{2}$–$10^{3}{\mu {m}}$ remains between the electrode and SGNs [19]. This physical separation has practical implications: stimulating current must traverse perilymph and the OSL bone to reach the SGNs, and the heterogeneity of this path is itself an opportunity. Whereas dense bone presents a high-impedance barrier that broadens current spread, the CPS pores (described in "Canaliculi Perforantes of Schuknecht (CPS)") are fluid-filled, low-impedance conduits along which current density is locally enhanced [19]. Closer electrode positioning (e.g., perimodiolar designs) reduces stimulation thresholds in part by exploiting these preferential paths [13]. Furthermore, the distance can vary along the cochlea: apical electrodes often achieve the smallest gaps $\approx {0.5}\text {mm}$ or less, while mid-cochlear electrodes can be $\approx {1}\text {mm}$ or more away [59]. These quantitative insights, drawn from human studies, validate the idea that CIs interface with the SGNs across a millimeter-scale gulf, rather than forming direct micron-level contact with the neurons. The same natural pores that conduct current preferentially are also potential highways for drugs, trophic factors, or neurites seeking the electrode array in the ST—a dual electrical/biological role that motivates aligning future biohybrid arrays with the CPS field [15, 16].

**Fig. 3:**
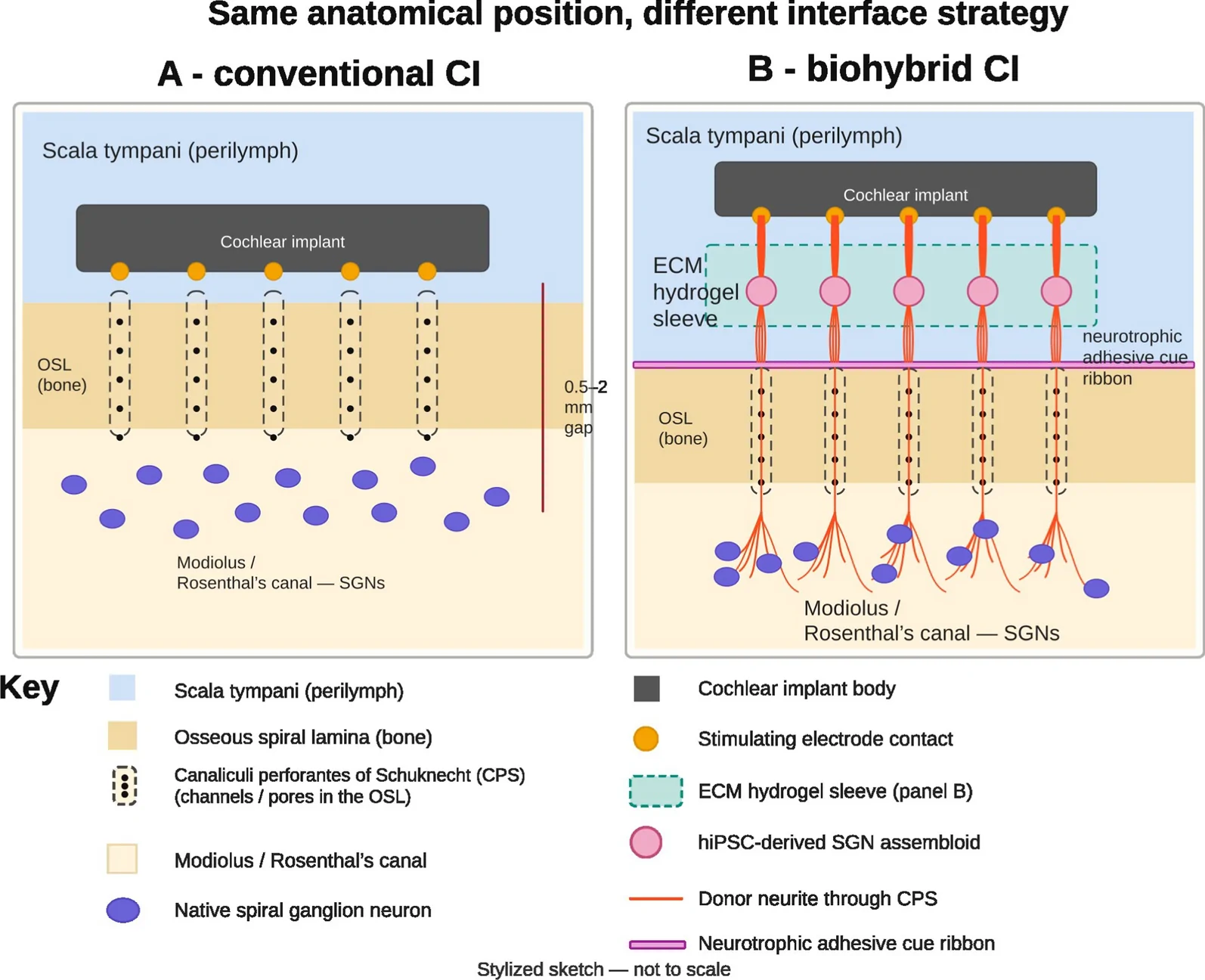
Conventional cochlear implant (CI) versus biohybrid CI: same anatomical position, different interface strategy. **A** Conventional CI. The implant body sits in the scala tympani (ST, light blue) along the modiolar wall; stimulating electrode contacts (orange) face the osseous spiral lamina (OSL, tan). The native canaliculi perforantes of Schuknecht (CPS, dashed channels with internal dots) traverse the OSL but only a fraction coincide with electrode contacts; current must therefore traverse perilymph and bone to stimulate spiral ganglion neurons (SGNs, blue ovals) in the modiolus/Rosenthal’s canal, across the intact electrode- neuron gap of typically 0.5–2 mm (red bracket). **B** Biohybrid CI. The implant occupies the same scala tympani trajectory along the modiolar wall, but each stimulating contact is paired with a hiPSC-derived SGN assembloid (pink) embedded in an extracellular matrix (ECM) hydrogel sleeve (dashed teal outline) and aligned with a CPS opening. Donor neurites (orange/red) extend from each assembloid through the CPS, traverse the OSL, and hypothesized to form a living bridge to native SGNs, recruited by localized neurotrophic, mechanical, and electrical cues; a neurotrophic adhesive cue ribbon (magenta line) at the ST–OSL boundary biases neurite ingress toward the CPS field. The contrast between A and B emphasizes that the biohybrid strategy preserves the conventional surgical approach while replacing the bulk-conduction interface with a cellular one–closing the electrode-neuron gap biologically rather than by relocating the electrode. Stylized sketch; not to scale

### Scala tympani dimensions and cochlear implant feasibility

#### Scala tympani anatomy in humans

Human (Homo Sapiens) As briefly mentioned in a previous section, the human ST is a large perilymph-filled canal that narrows from the base to the apex (Fig. 2A1 & A2). Near the basal turn (around the round window region), the ST cross-section is ovoid, with a typical vertical height of $\approx {1.2}{1.4}\, \text {mm}$ and a width slightly larger than [60]. This corresponds to a cross-sectional area of roughly ${2.3} \, {\text {mm}^{2}}$ at the base ($0^{\circ }$), which then diminishes to about ${1.3}{1.4}\,{\text {mm}^{2}}$ by $180^{\circ }$ (half a turn) into the cochlea [58, 60]. Beyond the basal turn ($\approx {360}{\circ }$), the ST lumen becomes more of a flattened triangle in cross-section as it enters the middle and apical turns. The height drops below 1 mm in the upper turns, and the area continues tapering (often $< {1}\,{\text {mm}}^{2}$ near the apex). Thus, the basal ST can accommodate larger diameters, whereas the apical region is very small and slit-like. Notably, the ST width is consistently greater than its height at any given point [64], reflecting the elongated shape of the duct. The human cochlea is $\approx {35}{36} \, \text {mm}$ in uncoiled length; when coiled it has 2.5–2.7 turns, and the total ST volume is $\approx {29}{\mu }\text {L}$ [65].

### Canaliculi perforantes of Schuknecht (CPS)

Schuknecht’s original temporal bone study (1959) revealed hundreds of microscopic channels piercing the OSL from the ST toward the modiolus [9]. Later histology and scanning electron microscopy confirmed that these canaliculi perforantes concentrate along the modiolar plate, especially in the basal and middle turns [9, 61–63, 66–68]. The CPS are predominantly located on the ST surface of the OSL, in the lower (inferior) bony lamina that underlies the basilar membrane. They are especially numerous in the modiolar (medial) region of the OSL, adjacent to the modiolus where the SGNs are housed [17] (Figs. 2B1, B2, C1 & C2 and  3). Human CPS lumina were measured from 0.2 to 27 *μ *m against an OSL thickness that tapers from 26.8 *μ *m basally to 8.4 *μ *m apically with local areal density $\approx 1.05 \pm 1.03$ pores per ${1000}{\mu }\text {m}^{2}$ (Fig. 2 C1, C2) [16, 17]. These dimensions overlap both the caliber of regenerating SGN neurites and the 10-30 *μ *m diffusion length of neurotrophins such as Neurotrophin-3 (NT-3) or Brain-Derived Neurotrophic Factor (BDNF) in perilymph, making each pore a matched conduit for axonal ingress and trophic support [69, 70].

### Other modiolar communication routes

[18] identified additional trabecular “meshwork”-like apertures within the modiolar trabecular network, measuring up to 100 $\mu \textrm{m}$ on the SV side and approximately 40 $\mu \textrm{m}$ on the ST side, with fenestrations along the perivascular and perineural sheaths. Together with the CPS, these openings establish a porous continuum that contradicts the notion of an impervious modiolar wall, permitting pressure equilibration and macromolecular traffic between perilymph and the SGN somata [17, 18]. Most recent fresh-frozen contrast-enhanced CT reproduced these soft-tissue corridors [15].

Additionally, the same investigators described “larger modiolar vascular outlets,” namely larger bony openings (up to $\approx {100}{\,\mu \text {m}}$) situated adjacent to the anterior and posterior spiral modiolar veins and arteries [17, 18]. The likely role of these vascular outlets is to permit exchange between the perilymphatic space and perivascular compartments, thereby contributing to perilymph homeostasis and offering potential routes for drug delivery [17, 18].

Complementing these channels, “mesothelial cell-covered fenestrae” were defined as thin cellular sheets ($< {3}{\,\mu \text {m}}$), lining the OSL and the modiolar surface, interspersed with micropores (up to $\approx {40}{\,\mu \text {m}}$) on both the ST and SV sides [17, 18]. Functionally, the mesothelial cell-covered fenestrae provide a semi-permeable lining over the bony channels, allowing diffusion of both small and large molecules while maintaining fluid compartmentalization; at the same time, by facilitating exchange between perilymph and perivascular/perineural spaces, they support perilymph homeostasis and represent potential conduits for local pharmacologic access [17, 18]. Table 1 summarizes quantitative dimensions of perilymph-modiolar passage channels in the basal turn of the human cochlea.

Furthermore, the retrocolumnar space (RS) (Fig. 2 C and D), the cleft behind the modiolar nerve columns from Rosenthal’s canal into the OSL, communicates with the modiolus through a wide trabecular lattice. In fresh tissue, it is lined by a thin (${0.3}{3}{\upmu }$) microporous mesothelial sheet over loose perivascular/perineural connective tissue; large modiolar apertures are present on the SV (to ${100}{\,\mu \text {m}}$) and ST (to ${40}{\,\mu \text {m}}$) sides. This architecture constitutes a low-resistance perilymph-modiolus interface and identifies the RS as a perimodiolar corridor that a biohybrid CI could leverage for micro-reservoir or microfluidic drug delivery and adjunct cellular constructs targeting SGNs [17, 18].

**Table 1:** Quantitative dimensions of perilymph-modiolar passage channels in the basal turn of the human cochlea

Structure / channel	Dimension (basal turn)	Source
Canaliculi Perforantes Schuknecht (CPS)	Mean diameter $5.2 \pm 4.9\ \mu$m (range 0.2- 27 *μ *m)	[16, 17]
Osseous spiral lamina (OSL) thickness	$26.8 \pm 6.0\ \mu$m	[17]
Density of canaliculi (pores per ${1000}{\mu }\text {m}^{2}$)	*1.05 ± 1.03* pores per ${1000}{\mu }\text {m}^{2}$	[17]
Mesothelial cell sheet thickness	0.3-$3\ \mu$m	[18]
Trabecular mesh fenestrae size	Up to $\approx 40\ \mu$m (holes in modiolar wall)	[18]
Modiolar vascular outlets	Pores up to $\approx 100\ \mu$m	[18]

CPS density values [17] are based on 2D pore counts per unit area in histologic sections. Recent 3D micro-CT of the osseous spiral lamina reports comparable pore-size distributions and regional enrichment near the modiolar plate, consistent with a porous OSL relevant to perilymph–modiolus communication [16]

### Distance between cochlear implant electrodes and spiral ganglion neurons in Rosenthal’s canal

Conventional CI electrode arrays reside in the ST and thus are separated from the SGNs in Rosenthal’s canal by the bony modiolus. Multiple anatomical and imaging studies confirm that across patients and electrode positions, the distance between an electrode contact and the SGNs in Rosenthal’s canal generally spans from a few hundred microns up to $\approx {1}{2}\text {mm}$ [71]. A detailed CT scan study of perimodiolar arrays reported a total range of $\approx {0.1}{1.8} \, \text {mm}$ in electrode-to-modiolus separation [10, 59]. Histological section measurements estimate that in the human basal turn, a lateral-wall electrode may be roughly *≈ * 2 mm away from the SGNs (by radial distance). These data support the statement that CI contacts lie within millimeters, but rarely mere microns of the SGNs [72].

The electrode-to-SGN distance is not uniform along the cochlea; it varies with the cochlear turn and electrode position. Overall, the closest electrode-neuron proximity is achieved in the apical cochlea, with the mid-cochlea generally being the farthest on average. However, patient-specific anatomy causes considerable variability (overall 0.1–1.8 mm range in the cited study) [59].

### The electrode-neuron gap does matter

[13] performed detailed histopathologic exams of five human cochleae with CIs and measured the distance between each electrode band and the center of Rosenthal’s canal. They reported that these electrode-to-SGN distances were within the millimeter range and, importantly, found a correlation between greater distance and higher electrical thresholds and comfort levels. In other words, electrodes located farther (several millimeters) from the SGNs required higher current levels to evoke hearing responses, reinforcing that distance is a key factor. The same study noted that intracochlear fibrosis or new bone formation could increase the electrode-modiolus distance, sometimes pushing contacts farther than their design intends. Nadol and colleagues, in histopathologic surveys, likewise estimated distances on the order of 0.5–2 mm and observed that any translocation of an array out of ST (or trauma to the modiolus) can reduce SGN counts, further emphasizing that the electrode-neuron gap matters [14, 73]. Computational models and anatomic maps further illustrate these distances. For example, a recent finite-element model by Sriperumbudur et al. examined electrical stimulation with a perimodiolar CI and assumed a *∼ *0.3 mm between the electrode and modiolus as a typical design [19]. This value ($300{\,\mu \text {m}}$) was chosen to reflect a snug perimodiolar placement; yet, it still highlights that even in simulation, the contacts are not assumed to touch the neurons (a 0.3 mm gap leaves space for the bony wall and perilymph) [19].

### Functional pathways and SGN connectivity through CPS

Distance alone, however, does not preclude effective interfacing if the intervening tissue is permissive. The CPS form a network of microscopic pores through the OSL that directly links the fluid of the ST with the perimodiolar spaces in Rosenthal’s canal [18]. By puncturing the lower plate of the OSL—alongside gaps in its mesothelial lining—and opening into the retrocolumnar trabecular meshwork and perivascular channels of the modiolus, these tiny canals create a continuous perilymphatic pathway from the ST into the SGNs. As a result, the extracellular fluid bathing the SGN cell bodies is essentially the same perilymph that fills the ST, permitting the free interchange of ions, nutrients, signaling molecules, and even therapeutic agents or pathogens between these compartments [15].

Although the true nerve fiber bundles use larger foramina (habenula perforata and modiolar conduits) to enter and exit Rosenthal’s canal, the CPS runs alongside and between those fiber routes, serving as perineural and perivascular fluid conduits. Glueckert et al. found nerve fibers in BDNF-treated animals that progressed into CPS. These fibers reveal a myelin layer close to the OSL and within the bony canaliculi, but exhibit extreme swelling upon entering the ST [74]. Similar observations have been reported by others [27, 75–77]. These findings provide direct anatomical evidence that endogenous SGN peripheral processes can traverse the CPS, supporting the use of the CPS field as a substrate for biohybrid bridging strategies (developed in Sects. "Selecting the bridge neuron: which cells belong in the CI–SGN gap?" and "Human stem-cell derived assembloids and hydrogel scaffolds for a living cochlear implant").

However, the swelling reported by Glueckert et al. signals that anatomical traversal alone is insufficient—neurites that successfully enter the CPS often degenerate as they cross the OSL-ST boundary. Several factors plausibly converge at this transition: loss of Schwann-cell-mediated myelin support beyond the OSL, altered perilymphatic ionic milieu, and BDNF-withdrawal-induced dysregulation of intrinsic growth pathways [74]. Three complementary mitigation strategies emerge from the literature and inform the biohybrid designs reviewed below: (i) sustained local NT-3/BDNF delivery via PODS^®^ or hydrogel depots to bridge the trophic gradient [78, 79]; (ii) ECM-matched hydrogel sleeves providing biomechanical and ionic continuity across the OSL-ST boundary [31, 80]; and (iii) modulation of intrinsic growth pathways—particularly PTEN/mTOR signaling, which regulates axon regeneration in central and peripheral neurons [81–85] and has been shown to control SGN neurite outgrowth and survival in the cochlea [86–88]. We note, however, that mTOR modulation must be carefully titrated, since both pro-survival and pro-degenerative effects have been described in the inner ear depending on context [89].

### Surgical implications for biohybrid CIs

*Surgical trade-offs of current arrays* Even before biohybrid components are considered, the mechanics of CI insertion impose constraints relevant to the modiolar interface. Insertion forces, electrode curl, choice of round-window versus cochleostomy entry, and depth of advancement all influence translocation rates, residual-hearing preservation, and intracochlear trauma [10–12, 90]. Because CPS density peaks in the basal OSL, traumatic insertion or aggressive drilling in this region risks sealing the very pathways that a biohybrid implant aims to exploit [15, 17]. Preoperative planning and post-operative assessment using modern 3D tools (e.g., OTOPLAN, ED slicer) increasingly support patient-specific trajectory planning that can minimize CPS-field disruption [91, 92].

*Biohybrid-specific constraints* A biohybrid CI does not bypass these insertion-mechanics issues; if anything, it amplifies them, because the implant carries cellular and hydrogel cargo that must survive the insertion event and remain positionally stable. Biohybrid arrays therefore must adopt atraumatic, compliant, shallow-insertion designs and avoid deeper modiolar drilling [7, 14]. Conversely, electrodes engineered with microfluidic ports or neurotrophin-releasing sleeves could harness the CPS pores to establish steep radial gradients that attract transplanted or residual neurites toward the contacts. Designing arrays that spare, rather than violate, the CPS therefore becomes as important as thread depth, pitch, or perimodiolar curl.

*Forward reference* Leveraging these natural conduits should reduce reliance on bulk diffusion, localize therapy to the modiolus, and ultimately tighten the electrode- neuron interface—a prerequisite for the cellular, materials, and surface strategies developed in Sects. "Selecting the bridge neuron: which cells belong in the CI–SGN gap?"–"Surface modifications to enhance neuron-to-electrode interactions". Future biohybrid CIs are also expected to incorporate microfluidic drug delivery to guide regeneration locally within the cochlea [24].

## Selecting the bridge neuron: which cells belong in the CI–SGN gap?

Having established that CPS-aligned pores and related modiolar conduits can be leveraged to shorten the electrode-neuron distance (Sect. "Anatomical rationale"; Fig. 2), we now ask which neuronal phenotype should occupy that space to enable a stable, synaptogenic interface. We emphasize that conventional CIs do not exploit the CPS: the pores are far too narrow (0.2–27 *μ *m) to admit a stimulating contact, and current electrode designs neither align with the CPS field nor deliver trophic or cellular cargo to its scala-tympani inlets [9, 17, 18]. Realizing the latent utility of these channels therefore requires a biohybrid design in which a bridge neuron, embedded in an appropriately tuned matrix, occupies the perimodiolar volume between the array and Rosenthal’s canal.

### What type of neurons belong in the gap?

The natural candidate is hiPSC-derived SGNs. However, strict auditory ontogeny may not be essential if the donor cells satisfy three functional criteria: (i) glutamatergic transmission compatible with *α *-amino-3-hydroxy-5-methyl-4-isoxazolepropionic acid receptor (AMPAR)-expressing SGNs [93], (ii) robust neurite extension guided by BDNF/NT-3 and inner-ear extracellular matrix (ECM—the network of secreted proteins, glycoproteins, and proteoglycans that surrounds cells and provides biochemical and biomechanical guidance) cues [72], and (iii) sufficient electrophysiological maturity for fast, phase-locked spiking [94]. In that light, hiPSC-derived glutamatergic neurons that are not explicitly patterned as SGNs could, in principle, act as relay cells when supported by the right scaffold and cue environment. Human cochlear organoid/assembloid models strengthen this premise by demonstrating ribbon-synapse features, neuritogenesis, and innervation in vitro [95, 96].

An additional functional criterion that follows from SGN biology is the requirement for high-fidelity temporal coding, which in vivo depends on Schwann-cell-mediated myelination of SGN peripheral and central processes. Type I SGN axons are myelinated with internodal spacing tuned to support sub-millisecond spike timing and phase-locking up to several kHz, and the heminode at the peripheral terminal serves as the spike-initiation zone–features that biophysical models show are essential for reliable propagation of synaptic input [97, 98]. Disruption of this myelination, even when transient, produces a permanent auditory-neuropathy phenotype in animal models, with reduced compound-action-potential amplitude, prolonged latency, and impaired temporal acuity [99]. The implication for biohybrid CIs is that hiPSC-derived bridge neurons must either (i) recapitulate Schwann-cell myelination—by co-delivering Schwann-lineage cells in the assembloid (Sect. "Human stem-cell derived assembloids and hydrogel scaffolds for a living cochlear implant")—or (ii) operate over short enough distances that unmyelinated conduction suffices for the relay function. Recent inner-ear assembloid systems already incorporate Schwann cells, making option (i) technically feasible [95, 96].

### Must the bridge neuron be SGN-lineage?

SGNs are specialized bipolar neurons with high-fidelity glutamatergic signaling, neurotrophin dependencies (BDNF/NT-3), and ion-channel compositions tuned for high-rate firing. Donor cells that recapitulate these attributes are most likely to integrate. In a large-animal model, hiPSC-derived cells delivered to deafened pigs migrated to the modiolar SGN region, increased SGN counts, and lowered CI stimulation thresholds—evidence consistent with functional auditory integration [100]. Engineered alternatives are also emerging: optogenetic strategies promise tighter spatial selectivity than electrical stimulation [101], and hiPSC-derived SGNs expressing channelrhodopsin have formed functional glutamatergic synapses with cochlear nucleus neurons in vitro [102]. Overall, SGN-lineage (or SGN-like) donors remain the most defensible choice, but well-specified, excitatory hiPSC neurons are a plausible fallback when they match SGN synaptic timing and trophic responsiveness.

### Will hiPSC-derived neurons synapse with endogenous SGNs?

Direct in vivo proof inside the human cochlea is limited; yet, several lines of evidence are encouraging. First, inner-ear assembloids–3D multicellular constructs that combine hiPSC-derived otic neurons with supporting glial and/or epithelial cells to recapitulate key features of the cochlear ganglion niche (described in detail in Sect. "Human stem-cell derived assembloids and hydrogel scaffolds for a living cochlear implant")—generate afferent-like synapses and SGN-compatible activity in vitro [95, 96]. Second, the adult cochlea shows some capacity for synaptic repair: blocking repulsive guidance molecule A (RGMa) facilitates the regrowth of auditory nerve synapses after acoustic trauma [103], and cochlear macrophages promote the reformation of IHC-SGN ribbon synapses and partial functional recovery [104]. We acknowledge, however, that this synaptic-recovery evidence concerns the IHC-SGN afferent synapse rather than direct SGN-SGN connectivity, and the donor-to-endogenous SGN interface envisioned in a biohybrid CI is therefore a novel synaptic configuration not directly supported by the cochlear literature alone. The more appropriate evidence base is the broader host-graft synaptic-integration literature in the spinal cord, cerebral cortex, and retina (see “Lessons learned from other systems” below), where transplanted human neurons routinely form functional synapses with host neurons of types they would not encounter in their endogenous developmental program. With proper placement near CPS inlets and the provision of neurotrophic and adhesive cues, donor SGNs are likely to form biologically plausible synapses onto residual SGNs, but this prediction must be tested empirically.

### Lessons learned from other systems

Cross-modal experience supports host–graft synaptogenesis and bidirectional relay formation. In chronic spinal cord injury, human neural progenitor grafts form reciprocal synapses with host neurons and restore meaningful function when combined with rehabilitation [105]. In the cerebral cortex, transplanted human cortical organoids integrate into thalamo-cortical circuits and influence behavior [106]. In the retina, stem-cell grafts form host–graft synapses that contribute to visual responses; recent work highlights specific roles for graft-derived horizontal cells in synaptic integration [107]. By analogy, a CI “bridge” neuron that is glutamatergic, synaptogenic, and neurotrophin-responsive should be capable of wiring into SGNs–particularly when guided toward the modiolus by CPS-aligned gradients.

### Pragmatic takeaway

If available, use otic-lineage (i.e., SGN) neurons because they align fastest with inner-ear cues and synaptic machinery. However, for the biohybrid CI gap, any mature, glutamatergic hiPSC neuron that satisfies synaptogenic and neurotrophin-responsive requirements is a defensible option—especially when paired with inner-ear-tuned scaffolds and pro-maturation programs.

## Human stem-cell derived assembloids and hydrogel scaffolds for a living cochlear implant

Biohybrid CI concepts rely on living neural elements (i.e. hiPSC-derived stem cells) that can mature, respond to therapy, and form or re-form synapses with endogenous SGNs [20]. In practice, this means (i) supplying the right cells in the right microenvironment and (ii) coupling them to the device through a mechanically and electrochemically compatible interface. In this section, we outline why three-dimensional (3D) stem-cell constructs—from simple spheroids to more complex assembloids—are preferred over dissociated cultures [108]. Recent inner-ear organoid/assembloid work has sharpened these arguments, reporting improved SGN-like phenotypes, synaptic specialization, and better translational relevance over 2D models [109, 110]. We will also discuss how xeno-free (i.e., free from nonhuman animal sources) hydrogels can provide a cochlea-ready niche that supports localized delivery and chronic electrical interfacing [111].

### Why 3D?—from spheroids, organoids to assembloids

Dissociated, two-dimensional SGN cultures show fragile survival and limited maturation [112], whereas 3D environments provide a pro-survival, pro-maturation niche with improved neuritogenesis, synaptic puncta, and electrophysiological properties closer to native SGNs [113–116]. Newer human cochlear organoid protocols report high-fidelity morphology and sensory–neuronal patterning that better match in-vivo benchmarks [96].

Spheroids (single-lineage aggregates) are easy to form and improve retention and trophic support after transplantation relative to single-cell suspensions [31]. A more recent synthesis emphasizes 3D micro-environments for SGN survival, neurite extension, and myelination [110]. Inner-ear *organoids* add rudimentary cyto-architecture and hair-cell structure to SGN ribbon synapses [114–116]. See also recent reports detailing synaptic specialization and long-term culture stability in inner-ear organoids [96]. *Assembloids* intentionally combine multiple, precisely chosen cell lineages to recreate elements of the SGN microenvironment. For example, SGN constructs that incorporate peripheral glia and vasculature–Schwann cells for axonal myelination, satellite glia for trophic/metabolic support of SGN somata, and a microvascular/pericyte compartment—are expected to mature more fully than neuron-only systems, achieving myelination with nodal specialization, improved neuronal health, and more adult-like excitability. Evidence includes robust myelination and node formation in human iPSC-neuron/Schwann co-cultures [117]; established trophic and homeostatic roles for satellite glia in sensory ganglia [118–120]; a direct survival role of pericytes for SGNs and the recognized need for vascularization in inner-ear organoids [121, 122]; and neuron-innervated cochlear organoids that better recapitulate the auditory unit than neuron-only constructs [95]. Additional PNS work shows glia-driven increases in neuronal excitability, supporting the maturation rationale [123].

Most recently, a four-factor small-molecule cocktail (GENtoniK; 1 *μ *M each of GSK2879552, EPZ-5676, NMDA,and BayK8644) accelerated the maturation of hPSC-derived neurons within one week, increasing neurite length, synaptic puncta density, and spontaneous action-potential firing while shifting transcriptomes toward postnatal, synaptic programs [124]. The emerging small-molecule maturation regimens (e.g., ’GENtoniK’) can further accelerate human neuron maturation in 3D systems; when used, these require pharmacological validation of synaptic readouts and careful dose scheduling to maintain viability and lineage identity [124].

### Design of SGN assembloids: cell composition and roles

A practical SGN assembloid includes four interacting cell types whose functions are complementary: (i) *SGNs* (afferent neurons, the principal excitable elements) [125]; (ii) *Schwann cells* (myelination distal to the habenula; metabolic and trophic support; debris clearance; immune orchestration) [96, 126]; (iii) *satellite glia* (ion and neurotransmitter homeostasis around somata) [127]; and (iv) *fibroblast/perineurial cells* (ECM deposition and compartmental definition) [128]. Recent comparative analyses of inner ear cell models and peripheral nerve micro-environments inform these lineage selections [109, 129]. We *deliberately omit a vascular compartment* in the intraluminal setting because the ST–facing bony wall lacks a perfusable vessel bed; expecting engineered endothelial–host anastomosis at this interface is unlikely and could promote fibrovascular responses [130]. The perineurial barrier and endoneurial microenvironment impose tight transport constraints and are not trivially re-created at the ST bony wall [129, 131]. Instead, one could use a diffusion-first design: compact ( *≤ * 300 *μ *m) 3D units embedded in a porous ECM so that oxygen and trophic diffusion distances remain within avascular limits while preserving alignment and myelination. Co-culture with a 3D microfluidic environment favors compact myelin formation around peripheral axons and higher-amplitude, more phase-locked firing, with evidence of ribbon synapses in innervated cochlear organoid systems [95]. See also organoid/assembloid systems that report robust SGN-like firing and synaptic markers [96].

Placement against the OSL, with fronts facing the CPS (Sect. "Anatomical rationale"), allows device-driven radial gradients (e.g., BDNF/NT-3) to recruit neurites through microchannels toward the modiolus rather than relying on the neovascularization of the ST wall.

#### Why target ${< 200 \mu m}$?

Avascular neural constructs become diffusion-limited for O$_2$ and macromolecular trophic factors once characteristic path lengths exceed *∼ *200 µm; beyond this, cores of hypoxia/necrosis are common unless the matrix is highly porous or actively perfused [31, 132–134]. Designing SGN assembloids as disks/ellipsoids with a smallest dimension < 150–200 µm (i.e., diameter < 200 µm if roughly spherical) keeps the half-thickness within avascular transport limits while still allowing sufficient cell numbers for myelination and phase-locked spiking when supported by Schwann cells [126, 135]. A porous, soft ECM further shortens effective diffusion distances (reduced tortuosity) and improves factor retention—important when shaping radial BDNF/NT-3 gradients from the device face toward CPS inlets (Sect. "Anatomical rationale") [20, 136, 137].

#### Why are small intraluminal assembloids appropriate in the scala tympani (ST)?

The ST is a bone-encased, perilymph-filled lumen lined by endosteum and lacks a perfusable capillary bed for grafted tissue; relevant vascular and perivascular spaces lie medial to the osseous spiral lamina within the modiolus [17, 18]. Although the OSL/modiolar wall contains canaliculi perforantes and mesothelial fenestrae that permit fluid exchange, these are not conduits for the rapid inosculation of host microvessels into the ST [18]. Consequently, intraluminal SGN assembloids must be engineered as avascular units supported by perilymph diffusion and device–proximal gradients. Diffusion-dominated transport in perilymph and extracellular tortuosity further bind usable length scales to $\mathcal {O}(10^2)$ µm [69, 70, 137]. Soft, neural-modulus hydrogels ($E \approx 0.5\text {-}3$ kPa) engineered with an interconnected macropore network (mode pore diameter 20–80 µm, void fraction *≳ *60%, inter-pore throat *≥ *10–20 µm) via cryogelation or microporous-annealed-particle (MAP) assembly are appropriate. These architectures shorten diffusion path lengths and lower tortuosity—improving effective diffusivity for O$_2$ and trophic factors—and provide channels large enough for SGN neurites and Schwann processes to infiltrate and align [70, 138–141]. Pursuing larger intraluminal constructs would require engineered perfusion (not present in ST) or pro-angiogenic strategies that risk fibrovascular ingrowth/new bone along the cochlear wall–findings associated with worse electrode–modiolus coupling [13, 73]. A conservative, diffusion-first design therefore best respects cochlear anatomy and transport limits [18, 69].

*Can intraluminal assembloids be “fed” trans-synaptically by endogenous SGNs?* In short, no—not at levels that substitute for a vascular supply. Synapses mediate neurotransmission and trophic *signaling* (e.g., BDNF/NT-3 capture and retrograde transport that aids survival and outgrowth) [30, 33, 75, 137], but synapses are not designed for bulk metabolite transfer (e.g., O$_{2}$, glucose). Long-range axonal energetics are sustained by glia and their vasculature-coupled metabolism: in the CNS, oligodendrocytes/astrocytes fuel axons via monocarboxylate transporters (MCT1/MCT2/MCT4) and glycolytic supply, and in the PNS, myelinating Schwann cells export lactate that axons use to maintain conduction and repair. Recent reviews and in-vivo studies reaffirm this glia-to-axon metabolic support paradigm [142–145]. With no perfusable bed in the ST and perivascular networks confined to the modiolus [17, 18], intraluminal SGN assembloids should be treated as avascular: keep the smallest dimension < 150–200 µm, use porous soft ECMs, and supply short-range device-proximal gradients (e.g., BDNF/NT-3) toward CPS inlets [20, 69, 70, 133, 137–139].

#### Porous ECM for SGN assembloids: why laminin, and what to pair it with?

As we discussed in our previous section, because the ST wall provides no perfusable vessel bed, we do not include a vascular compartment in intraluminal SGN assembloids. Instead, viability and function should be secured by (i) keeping constructs *≤ * 200 µm in their smallest dimension [146], (ii) embedding them in a porous, neurite-permissive ECM, and (iii) driving *radial* trophic gradients from the device face toward CPS mouths (Sect. "Anatomical rationale") [20, 136, 137, 147]. Particularly, neurite-permissive, porous ECM is crucial for neuronal viability and function. By enriching the matrix with neural-specific ECM components (e.g., laminin), neurons receive proper integrin-mediated signals, leading to better neurite outgrowth and cell survival [148].

Why laminin as the spine for biohybrid CI? Laminins are the dominant basement-membrane ligands for both SGNs and Schwann cells; they engage laminin-binding integrins to promote neurite extension, Schwann cell alignment, and myelination [149]. In peripheral nerve and basal lamina-like matrices, laminin isoforms consistently bias outgrowth and support compact myelin while reducing the need for non-specific Arginylglycylaspartic acid (RGD) cues that can recruit fibroblasts [149, 150]. Recombinant human laminin-511/521 (or laminin-derived peptides e.g., Ile-Lys-Val-Ala-Val [IKVAV] and Tyr–Ile–Gly–Ser–Arg [YIGSR]) provides a defined, xeno-free backbone that is highly neuritogenic and compatible with GMP manufacturing [151]. A more recent review highlights laminin-511/521 as the preferred xeno-free matrices in clinical-grade hPSC systems [152–154].

Other Extracellular Matrices Collagen IV complements laminin to form a basement membrane-like network that SGNs and Schwann cells recognize; it stabilizes laminin and provides gentle scaffold strength without imposing high stiffness [155–157]. Recent overviews of Schwann-cell–ECM dynamics and peripheral-nerve matrices support laminin-biased, low-stiffness blends for neurite guidance [129]. Collagen I, in contrast, forms fibrillar, typically stiffer gels that are broadly pro-adhesive (RGD-rich) and can promote fibroblast infiltration [158–161]. In an intraluminal niche where fibrosis is undesirable, a laminin + collagen IV matrix can be more favorable; if collagen I is used at all, it must be kept dilute and blended with laminin (and/or hyaluronan) to maintain a low bulk modulus that is neurite-friendly [157, 162]. Vitronectin and fibronectin–RGD-rich proteins are excellent for hiPSC maintenance and general adhesion, but they are less specific for SGN/Schwann programs and can potentiate fibroblast attachment. Use them sparingly (e.g., as micro-patterned adhesion islands for initial handling) or omit them in favor of laminin-biased ligands (IKVAV, YIGSR) when the goal is long-term neuronal alignment and myelination [149].

Porosity and mechanics. To prevent core necrosis without perfusion, the ECM should match brain/nerve-like moduli (*≈ *0.5–3 kPa) and incorporate *intrinsic microporosity* to shorten diffusion paths and lower tortuosity [161, 163, 164]. Recent cryogel reviews and microporous-hydrogel advances (granular/annealed particle) provide practical routes to cell-scale porosity at neural-range moduli [165–169]. Practical routes include sacrificial gelatin/alginate microspheres or microfibers yielding interconnected 20–80 µm pores, cryogelation to form supermacroporous, highly permeable networks, and microporous-annealed-particle (MAP) gels assembled from microgels (“annealed microgels”) for cell-scale porosity and rapid mass transport [138–141]. Hyaluronan-rich blends further increase water content, reduce matrix friction, and support neurite outgrowth when combined with laminin cues [141, 164]. Geometrically, keeping the smallest spheroid axis *≲ *150–200 µm respects avascular diffusion limits for O$_2$ and macromolecular trophic factors [70, 133], which should be verified empirically (live–dead/hypoxia reporters; physiology) in static culture.

### Hydrogel scaffolds as the SGN assembloid niche

The clinical translation of a bio-hybrid cochlear implant hinges on a three-dimensional scaffold that is *xeno-free*—to eliminate batch-to-batch variability, undefined growth factors, and potential zoonotic agents intrinsic to matrices such as Matrigel. Candidates should possess self-assembling peptides, which are short synthetic peptides that spontaneously organize into nanofibers and form 3D hydrogels used as ECM-mimicking scaffolds for future biohybrid CIs. Finally, hydrogels are logical load-bearing supports for any membrane-mimetic interfacial layers on CI electrodes [24]. Among various hydrogels (see Table 2), we will discuss the two most promising hydrogels pertinent to our biohybrid CI designs.

#### VitroGel^®^ NEURON

VitroGel^®^ NEURON is a fully synthetic, xeno-free polysaccharide hydrogel that gels at room temperature by ionic exchange (no thermal or chemical initiators) [170, 171]. Ready-to-use formulations show a storage modulus of *∼ *100–300 Pa (manufacturer rheometry), corresponding to a Young’s modulus of *∼ *0.3–0.9 kPa [172]. The system is supplied with compatible peptide-functional VitroGels (RGD, IKVAV, YIGSR) to dial in neurite-permissive cues [173–175]. Its transparent, physically cross-linked network supports shear-thinning and self-recovery typical of ionic polysaccharide gels—useful for handling and dissipating micro-strain—while maintaining imaging compatibility [171, 176, 177]. Note that independent peer-reviewed characterization for neuron cultures remains limited, so we report the vendor’s rheology and kit specifications here [170–175].

#### PeptiGel^®^ Alpha 4 vs. Alpha 4 PLUS

We also focus here on PeptiGel^®^ Alpha 4 PLUS as a default SAP scaffold near cochlear-implant electrodes because it preserves the soft Alpha 4 mechanical profile (vendor rheometry: $G'\!\sim$0.7–1.3 kPa) and net positive charge while introducing fibronectin-mimetic RGD and collagen-mimetic GFOGER motifs to engage integrins that support neural adhesion [159, 178–180]. By contrast, Alpha 4 lacks these built-in adhesion cues and is best framed as a platform for custom laminin-derived tethering (e.g., IKVAV/YIGSR) when SGN-specific guidance is desired [149, 181]. The auditory literature consistently indicates that laminin-biased cues (full-length laminin or its peptide mimetics) promote SGN neurite extension and directional guidance more effectively than generic RGD alone [80, 149, 182–185]. In practice, either (i) Alpha 4 PLUS can be used “as supplied” when broad integrin engagement is desired at the device face, or (ii) Alpha 4 can be functionalized with laminin-derived sequences to bias SGN/Schwann programs in a more lineage-specific manner, keeping the bulk modulus in the neural range. See Table 2 for a summary of all six candidate animal-free hydrogels evaluated against these criteria.

**Table 2:** Comprehensive comparison of candidate animal-free hydrogels for the hiPSC-derived SGN assembloids niche

Hydrogel (supplier)	Composition and salient features	Pore / mesh size	Young’s *E* (kPa)
VitroGel^®^ NEURON (The Well Bioscience)	Fully synthetic, xeno-free polysaccharide network; shear-thinning; customizable peptides	Nanoporous (*≈ * 200–500 nm pore size)	0.3–0.9
PeptiGel^®^ *α *4 PLUS (Cell Guidance System)	Self-assembling *β *-sheet peptide; pre-functionalized with adhesive SAP (RGD and GFOGER)	*≈ * 100 nm fibers	0.7–1.3
PuraMatrix^®^ (Corning)	Ionic RADA16 peptide nanofibrils; > 99% water; amphoteric surface	50–200 nm fibers	0.1–1
RGD-alginate (NovaMatrix FMC)	$Ca^{2+}$-crosslinked alginate grafted with RGD; enzymatically degradable	< 5 nm mesh	3–20
HyStem-HP (Sigma)	Thiolated HA + gelatin + PEGDA; heparin-binding; tunable gel time	*≈ * 17 nm mesh	0.3–2
PEG-DA hydrogels (Academic)	Photocrosslinkable PEG network; inert unless RGD-modified	5–10 nm mesh	0.5–20

hiPSC = human induced pluripotent stem cell(s); SGN = spiral ganglion neuron(s); RGD = Arg–Gly–Asp integrin-binding motif; GFOGER = Gly–Phe–*O*–Gly–Glu–Arg (where *O* denotes 4-hydroxyproline); HA = hyaluronic acid; PEGDA/PEG-DA = poly(ethylene glycol) diacrylate; RADA16 = Ac–(Arg–Ala–Asp–Ala)_4_–NH_2_ (self-assembling peptide)

In summary, a minimal design rule emerges: choose a xeno-free, soft (0.5–3 kPa), peptide-adhesive gel that can be injected and quickly recover, then decorate with ECM motifs (RGD/IKVAV/YIGSR) and gradients of neurotrophins (e.g., BDNF/NT-3) to bias neurite trajectories. In several cochlear and neural contexts, peptide-functionalized hydrogels attract SGN neurites and support long-term survival. In inner-ear models, nanofibrillar cellulose and related systems have been used to create a 3D niche with sustained factor release [31, 80, 110, 186]. In these works, hiPSC-derived SGN assembloids offer a controllable, multi-lineage route to a living CI interface, while modern, xeno-free hydrogels supply the compliant, bioactive niche and delivery vehicle that those cells need. When combined with conductive and anti-fouling coatings, these materials have the potential to narrow the effective electrode-neuron distance and maintain implant performance while enabling regeneration-driven improvements over time in the future [20, 22, 31].

## Biomaterial interfaces for next-generation cochlear implants

Biohybrid "living" cochlear implants must balance three coupled demands: (i) efficient charge transfer at the metal-tissue boundary, (ii) mechanical and electrochemical compliance to match inner-ear tissues while maintaining stable stimulation and recording, and (iii) durable resistance to fibrosis, infection, and biofouling. A practical material stack proceeds from established noble-metal contacts to conductive polymer interlayers, then to conductive hydrogels and anti-fouling chemistries that enable regenerative and drug-delivery functions [24].

### Platinum-Iridium (Pt-Ir): the clinical baseline

Platinum-iridium (Pt-Ir) remains the workhorse for intracochlear electrodes due to its corrosion resistance and long clinical track record. Chronic stimulation studies continue to refine safe operating windows and dissolution limits, and thin-film variants have been characterized electrochemically and in vivo [7, 187, 188]. Pt-Ir establishes a reliable, inert foundation but offers limited mechanical compliance and comparatively modest charge-injection capacity relative to newer coatings.

### Indium tin oxide (ITO) in living biohybrid interfaces

ITO is an optically transparent conductive oxide long used to make transparent microelectrode arrays (MEAs) [189], enabling simultaneous optical interrogation (e.g., imaging or optogenetic actuation) and electrical readout of living neural cultures [189–191]. For a biohybrid CI concept, that transparency can be leveraged during ex-vivo assembly/testing of SGN assembloids on the array (morphology, Ca$^{2+}$ imaging) and in optogenetic prototypes, to minimize photoelectric artifacts while preserving electrical access [191, 192]. The main liabilities of ITO are (i) higher impedance/noise than classic neuroelectrode materials and (ii) brittleness upon flexion; recent studies, therefore, use ITO primarily for transparent traces/windows and add low-impedance micro-islands (PEDOT, IrOx, or ultrathin TiN) at the actual stimulation sites [193, 194]. Practically, this hybrid stack retains optical access to living tissue while restoring electrochemical performance to neuroprosthetic norms. Where neurite guidance is desired, ITO and overcoats can be further functionalized with laminin-biased cues (full-length laminin or IKVAV/YIGSR peptides); in vivo work with laminin-coated CI electrodes has shown enhanced neurite targeting and lower ABR thresholds, underscoring the value of laminin chemistry at the interface [195, 196].

### Conductive polymers (CPs)

Conductive polymers such as PEDOT:PSS and polypyrrole (PPy) lower impedance and increase charge injection capacity relative to a bare Pt–Ir electrode, improving stimulation efficiency at neural interfaces [197, 198]. CP coatings can be photo-patterned and, when covalently anchored to metals (e.g., via aryl–diazonium or iodonium primers), exhibit improved adhesion and aqueous stability [199–203]. Beyond electrochemistry, PPy matrices loaded with NT-3 or BDNF promote spiral-ganglion neurite outgrowth in preclinical models [147, 204]. Overall, CPs offer a mature route to reduce impedance and incorporate biomolecules, though long-term cohesion/adhesion under micromotion remains a concern [205, 206].

### Conductive hydrogels (CHs)

CHs combine tissue-like mechanics with mixed ionic/electronic transport, reducing interfacial impedance and improving coupling [207]. In cochlear systems, CH coatings on clinical-style arrays improved electrochemical performance in bench/device tests, and in vivo stimulation studies reported higher charge storage capacity (CSC) and charge injection limit (CIL) and lower voltage-transient impedance than Pt while maintaining neural function, though some coating loss was noted [188, 208]. Practically, a thin PEDOT pre-layer or surface priming is frequently used to secure hydrogel coatings to metal contacts [188, 201, 202]. Because the hydrogel phase can host cargos, CHs also enable the local delivery of anti-inflammatory or neurotrophic agents (e.g., BDNF and NT-3) at the electrode–neuron interface [209, 210].

### Polyimide–parylene-C encapsulation of flexible CI arrays

Polyimide (PI) is a widely used flexible substrate for thin-film cochlear implant (CI) arrays, and Parylene-C (Pa-C) is a common conformal biocompatible overcoat and moisture/ion barrier. Recent encapsulation studies emphasize (i) Pa-C’s low permeation relative to many polymers but vulnerability to defect-driven ingress (pinholes/microcracks) and interfacial delamination, particularly around topography and edges; (ii) the importance of adhesion promoter and surface activation steps (e.g., A-174 silane, O$_2$ plasma, annealing) to stabilize PI/Pa-C and metal/Pa-C interfaces; and (iii) inorganic–polymer dyads (e.g., atomic layer deposited (ALD) Al$_2$O$_3$/Pa-C) that mitigate defect pathways and extend durability in Reactive-Accelerated Aging (RAA) soak and, in some cases, in vivo tests [211–215].

In flexible neural packaging, Pa-C/ALD nanolaminate dyads generally outperform single-layer Pa-C in accelerated aging and device-level evaluations; PI remains a robust mechanical backbone when coupled to a chemically bonded Pa-C overcoat [212, 215–218]. These stacks are compatible with electroactive (PEDOT/PPy), conductive-hydrogel, and zwitterionic topcoats, provided that adhesion is engineered at each interface and sterilization conditions are validated so that thin inorganic sub-layers (e.g., Al$_2$O$_3$) are not damaged [34, 201–203, 213].

### Poly(*\varepsilon *-Caprolactone): structural carriers and drug depots

Poly(*\varepsilon *-caprolactone) (PCL) is a semicrystalline aliphatic polyester (melting point *≈ *59- 64$^{\circ }$C) with a glass-transition temperature near *-56* to $-60^{\circ }$C, which allows it to remain flexible at 37$^{\circ }$C. It is widely used in FDA-cleared devices and has an FDA material safety summary for implanted use [219].

Porous PCL architectures are readily obtained by supercritical CO_2_ foaming, yielding highly interconnected macropores (typical pore size *∼ *70–180 *μ *m, porosity *∼ *90%, interconnectivity *∼ *96%), which are appropriate for neural tissue ingrowth and mass transport. By contrast, standard electrospun PCL mats, while highly porous, usually present sub-tens-of-micron pores; achieving cell infiltration generally requires pore-enlarging strategies [220].

The tensile modulus of PCL strongly depends on porosity. Bulk PCL typically exhibits $E\approx 3.4\times 10^{2}$ MPa, whereas highly porous PCL scaffolds fall in the *∼ * 35–140 MPa range, compatible with use as a compliant mechanical buffer between soft hydrogel compartments and rigid device elements [221].

Hydrolytic degradation proceeds slowly (months to years) via ester-bond scission, producing oligomers and 6-hydroxyhexanoic (6-hydroxycaproic) acid. Relative to PLA/PLGA, PCL’s products are less acidifying and show reduced autocatalysis; moreover, small solutes of this size are cleared from perilymph on time scales of tens of minutes to hours, limiting sustained pH excursions in the cochlea [222, 223].

These properties align with PCL as an intermediate layer that (i) mechanically buffers the living construct from metallic features, (ii) provides macroporous reservoir volume for sustained-release cargos (e.g., PODS^®^ neurotrophin co-crystals), and (iii) supports topographical guidance. Sustained neurotrophin delivery with PODS has been demonstrated in neural contexts, and topographical cues–aligned fibers or micro-/nano-patterned surfaces–direct spiral ganglion neurite orientation in vitro [31, 224].

Recent work using fibrin-coated electrospun PCL demonstrates that a thin ECM overlayer mitigates PCL hydrophobicity, improves cell retention, and supports hiPSC-derived neural progenitor differentiation to functional dopaminergic neurons with microelectrode-array activity; mechanical testing of the coated mats remains in the tens-of-MPa regime [225].

### Anti-fouling and anti-inflammatory surface chemistries

Antifouling surface chemistries are now being applied directly to CI carriers and arrays. Photografted zwitterionic thin-film hydrogels (e.g., pCBMA/pSBMA) covalently attached to PDMS on human cochlear-implant arrays reduced subcutaneous fibrotic capsule thickness over 6–52 weeks in vivo, indicating a durable reduction of the foreign-body response (FBR). Complementary durability testing shows large decreases in tissue–implant friction, reduced insertion forces in cadaveric cochleae, and robustness to bending and transient desiccation [34, 226]. Recent intracochlear animal work with zwitterion–polydopamine coatings further reports reductions in macrophage activation, fibrosis markers (e.g., *α *-SMA/collagen I), and better residual-hearing preservation [227].

In parallel, steroid-eluting electrode strategies have progressed from rodent and NHP models to human feasibility and early clinical experience. Across preclinical models, dexamethasone-eluting arrays consistently reduce fibrosis and inflammatory responses and lower electrode impedance; human studies report lower and more stable impedances compared with standard arrays, while a systematic review indicates that hearing preservation and safety outcomes in humans are still being established [228–232]. Steroid delivery via a molded round-window niche implant presents another localized route under investigation [233].

Together, antifouling chemistries and controlled-release approaches are complementary to CP/CH electrode stacks: they aim to blunt the peri-implant FBR (macrophage infiltration, fibroblast activation, fibrous encapsulation) while electroactive layers tune the electrode- tissue interface. [34, 226] Anti-biofilm chemistries and long-term moisture/ion encapsulation barriers, which address overlapping but distinct failure modes (microbial colonization and dielectric drift), are discussed in Sect. "Approach 4: Anti-biofilm chemistry and long-term encapsulation barriers" as part of the surface-modification framework for biohybrid arrays.

### Sustained neurotrophin delivery and neurite guidance

In the past decade, substantial progress has been made in delivering neurotrophic therapy to the inner ear [33]. Viral vector-based gene delivery (AAV, Ad28) enables long-term localized expression of BDNF in supporting cells or SGNs. Modified promoters can minimize adverse effects from overexpression while maintaining therapeutic protein levels [234, 235]. Hydrogel and nanoparticle depots—such as biodegradable hydrogels and nanoporous silica—provide controlled, sustained release of BDNF over weeks without requiring continuous infusion, thereby improving protein stability and SGN preservation [110, 236, 237]. TrkB-agonist mimetics, including small molecules like 7,8-dihydroxyflavone or monoclonal antibodies, mimic BDNF effects while offering better pharmacokinetic stability and diffusion, bypassing issues related to large protein delivery with a proven ability to promote SGNs survival and synaptic repair ex vivo [238]. Cell-based coatings on electrode arrays using BDNF-secreting cells offer localized, on-site neurotrophic support, preserving SGNs and enhancing neurite regrowth in animal models [30]. Together, these strategies provide spatiotemporal control of BDNF delivery, balancing efficacy with safety for clinical translation.

#### The polyhedrin delivery system (PODS^®^)

PODS^®^–BDNF consists of BDNF co-crystallized within a polyhedrin protein lattice to form stable, micron-scale crystals [239–244]. These depots resist dissolution and instead release BDNF as the lattice undergoes slow, local proteolysis, generating sustained, quasi–zero-order release over weeks—an attractive counterpoint to the minute-scale systemic half-life of soluble BDNF (plasma *∼ *110 min; CSF *∼ *60 min) [245–247]. In cochlear-relevant models, a longitudinal “neurotrophic strip” or gradient along the array is feasible using PODS embedded in sleeves/coatings, continuously bathing nearby SGNs [20, 31]. Manufacturer stability data further indicate long-term preservation of bioactivity within the crystalline state (e.g., *∼ *70% activity at 6 months at $37^{\circ }C$) [248].

Compared with alternatives, PODS targets the core delivery problems without introducing living cells or gene expression: AAV-BDNF can achieve longevity but is hard to confine, and overexpression can be maladaptive in normal cochleae [249]; hydrogel or thin-film depots often show early bursts and taper within days to weeks [250]; nanoparticles/microspheres extend release but commonly retain an initial burst and incomplete elution [186]; small-molecule TrkB agonists diffuse well but have short half-lives and have underperformed BDNF in preserving SGNs and function [251, 252]; and encapsulated cell therapies add surgical and safety complexity, with benefits limited to the implant period [253]. By contrast, PODS–BDNF integrates directly with polymer/hydrogel carriers (e.g., PCL sleeves, conductive hydrogels) without loss of activity, maintains the mechanical integrity of the composite, and supports device-aligned gradients to recruit and sustain neurites at the interface [20, 31]. Note that brand names are used for identification only; no endorsement is implied; all trademarks are the property of their respective owners. Also note that our translational approach does not place living cells on or affix them to electrode faces; instead, living constructs are positioned in adjacent ECM sleeves to leverage native porous pathways.

## Surface modifications to enhance neuron-to-electrode interactions

A persistent barrier to CI performance is the sub-mm electrode- SGN separation, which increases current spread and reduces channel selectivity. In a biohybrid CI, surface engineering can (i) lower the electrochemical barrier, (ii) guide neurites toward contacts, and (iii) suppress early fouling and chronic inflammation that elevate impedance. Below are four complementary approaches.

### Approach 1: microstructured electrode surfaces

Microscale topography can bias SGN neurite alignment and turning via growth-cone mechanosensation. Ridge-groove patterns on the order of a few micrometers in period and depth have been shown to promote alignment in neural models, and reviews detail how such topographies interface with neurons [254, 255]. In vivo, microstructured CI electrodes have exhibited the expected trade-off: while structures can influence tissue responses, elevated features increased cochlear damage and impedances in a guinea-pig model, underscoring the need for shallow/rounded features and careful insertion mechanics [256]. More generally, literature on penetrating microelectrodes highlights the tissue damage risks from sharp protrusions, supporting the use of compliant overcoats and gentle profiles [257]. Practically, low-aspect-ratio structures embedded in a compliant coating are ideal, especially when present with permissive ECM ligands in the coating (e.g., laminin motifs) to amplify guidance without adding stiffness discontinuities [209].

### Approach 2: conductive and electroactive coatings

Roughened noble metals and conducting polymers (CPs) such as PEDOT and PPy reduce interfacial impedance and increase charge-injection capacity relative to bare Pt/Pt–Ir [197]. Conductive hydrogel (CH) coatings on commercial CI arrays further increased charge injection and maintained reduced impedance through extensive pulsing [208]. Under chronic intracochlear stimulation, CH-coated electrodes retained electrochemical advantages, though some coating loss was observed–making adhesion and sterilization compatibility key design checks [188]. CP/CH systems also support biofunctionalization and factor delivery (e.g., PEDOT with RGD-alginate enabling sustained BDNF delivery and improved CI function) [209]. Early mechanistic work on CP microstructures provides additional context for tailoring electrochemical performance [258].

### Approach 3: antimicrobial and pro-healing interfaces

Polydopamine (PDA) is a widely used primer that enhances adhesion and enables peptide presentation. On CI silicone carriers, PDA–peptide films increased hydrophilicity and adipose-derived stem-cell adhesion without raising insertion forces [259]. Zwitterionic chemistries grafted onto CI-relevant materials reduced protein/cell adhesion and showed antibacterial and anti-inflammatory effects in vivo in rat models [227]. These strategies are suited to the tissue-facing surface, while underlying electroactive layers handle charge transfer.

### Approach 4: anti-biofilm chemistry and long-term encapsulation barriers

Anti-fouling zwitterionic chemistries that suppress the conditioning protein film and reduce postoperative inflammation are described in Sect. "Anti-fouling and anti-inflammatory surface chemistries" and are not repeated here. Where sustained antibacterial action against bacterial colonization is required—a distinct failure mode from foreign-body fibrosis—NAC-functional polysiloxanes provide antibiofilm behavior while remaining compatible with elastomeric carriers [260]. For long-term moisture/ion barriers on flexible arrays, polymer- inorganic dyads (e.g., ALD Al_2_O_3_/Parylene-C or AlOx-Pa-C stacks) mitigate defect-driven transport and extend lifetime in accelerated testing, and are compatible with the PEDOT/CH and zwitterionic topcoats described above [216].

## Conclusions and translational outlook

Modern cochlear implants restore audibility for hundreds of thousands of users, yet an enduring electrode–neuron separation limits spatial selectivity and temporal fidelity. Converging evidence suggests that anatomically guided, multimodal interfaces can narrow this functional gap: (i) localizing biochemical and electrical cues near the modiolus to confine current spread; (ii) using compliant, low-impedance stacks (conductive polymers/conductive hydrogels with anti-fouling chemistry) to stabilize charge transfer at tissue-like modulus; and (iii) adding on-demand micro-transport and patterning where power/thermal budgets permit [4, 7, 188, 197, 261, 262].

### Compact 3D neural units as a general pathway

Compact 3D neural units as a general pathway. A broadly applicable direction is the use of compact, diffusion-first human iPSC–derived SGN assembloids embedded in porous, neurite-permissive matrices and oriented toward modiolar microchannels. Recent work shows accelerated maturation programs for hPSC-derived neurons and balanced appraisals of inner-ear organoids’ translational promise and constraints [124, 263]. In parallel, acoustics-enabled biofabrication offers contact-free handling and patterning of delicate 3D aggregates [264].

### Evidence package and boundaries

Before a first-in-human feasibility study, a credible dossier should include: (1) reproducible demonstration of modiolus-adjacent accessibility and local gradient control in adult anatomy; (2) quantifiable neurite bias in anatomically faithful preparations under combined trophic, mechanical, and electrical cueing; (3) interface-level gains in vivo (ECAP/eABR thresholds, SOE width, channel interactions) with longitudinal impedance/noise tracking [265–269]; (4) atraumatic insertion with familiar surgical workflows [11, 12, 90]; and (5) chronic stability of materials/coatings in perilymphic conditions [215–217, 270].

### Field-level needs

Shared datasets (adult modiolus-adjacent maps; standardized transport/field models; harmonized ECAP/SOE protocols) and long-duration materials data in cochlear conditions would enable apples-to-apples comparisons. If the anatomy-plus-function criteria above are met with durable performance and acceptable risk, biohybrid cochlear interfaces merit staged clinical evaluation.

## Supplementary Information

Supplementary Material 1.

## Data Availability

No datasets were generated or analysed during the current study.
