# Supplementary material for: Biohybrid cochlear implants: neural interfaces, regenerative pathways, and translational benchmarks
Source: J Neuroeng Rehabil. 2026 Jun 30;23:278. doi: 10.1186/s12984-026-02069-5 (PMC13591839; doi:10.1186/s12984-026-02069-5)
Supplement: Supplementary file 1 [file 12984_2026_2069_MOESM1_ESM.pdf]

Supplementary Information

Table S1: Representative patent families relevant to biohybrid/living cochlear-implant interfaces and enabling technologies (non-exhaustive, descriptive only).

Focus area	Representative filing (jurisdiction, number, year)	Assignee / Applicant	Key idea	Notes / status
**Biohybrid CI with cells on electrodes**	US 9,265,933 (2016); US 2007/0093878 A1	Massachusetts Eye & Ear (MEEI)	CI electrodes *carrying viable neural progenitor cells*; processes bridge to neural targets to improve coupling.	Granted (US).
**"Living electrodes" / tissue-engineered constructs**	WO 2017/181068; US 2021/0393960 A1; US 12,090,323 (2024)	The Trustees of the Univ. of Pennsylvania	Micro-TENNs: ECM core + hydrogel sheath with neuronal aggregates; biological conduits for stimulation/recording.	Granted (US 2024); family active.
**Biohybrid device (living cells on flexible MEA)**	WO 2024/133777 A1 ("Biohybrid medical device")	Cambridge Enterprise Ltd.	Flexible PEDOT:PSS/parylene arrays integrated with iPSC-derived cells (e.g., myocytes for peripheral-nerve interfacing) at electrode sites for chronic, stable bioelectronic coupling.	PCT published 2024; pending.
Drug-eluting CI (early concepts)	US 2007/0213799 A1	MED-EL	Electrode array with drug-eluting portion (e.g., steroid) via silicone rods/slots/layers for intracochlear release.	Abandoned (US) — historical prior art.

Continued on next page

Focus area	Representative filing	Assignee / Applicant	Key idea	Notes / status
Drug-delivery accessory / sleeve	US 8,617,097 (2013)	Cochlear Ltd.	Tubular drug-delivery sleeve or tip attachment that compresses onto an elongate implant (e.g., a CI array).	Granted (US).
Maintain low electrode impedance	US 7,519,435 (2009); US 8,175,722 (2012)	Cochlear Ltd.	Anti-adhesion coatings (e.g., PEG) and electrochemical cleaning protocols to control chronic impedance and interface health.	Granted (US).
Mechanical isolation of contact-tissue interface	US 11,738,193 (2023)	Cochlear Ltd.	Raised mechanical barrier (continuous silicone lip) extending around each electrode contact, urged against tissue to inhibit body-fluid ingress and define a sealed contact volume.	Granted (US).
Mechanically anchored hydrogel coverings on contacts	EP 3041444 B1; US 10,870,001 (2020)	MED-EL	Biocompatible hydrogel layer fixed over electrode contacts *solely by mechanical connection* (loops through openings, fixation grooves) that swells in perilymph to minimize fibrous encapsulation.	Granted (EU/US).

Continued on next page

**Focus area**	**Representative filing**	**Assignee / Applicant**	**Key idea**	**Notes / status**
Hydrogel-filled drug reservoirs in array	US 8,750,988 (2014)	MED-EL	Drug lumen within the electrode carrier with a hydrogel matrix between lumen and delivery openings, controlling diffusion of therapeutic fluid (e.g., dexamethasone) into the cochlea.	Granted (US).
Dexamethasone coating process (CI carrier)	US 10,772,996 (2020)	MED-EL	Solvent/non-solvent silicone coating process embedding dexamethasone for sustained release to the perilymph.	Granted (US).
Bioresorbable coating with exposed contacts	US 7,949,412 (2011)	Advanced Bionics AG	Bioresorbable coating on the medial side of the array (PLLA/PLGA/PCL/PHB) leaving metal contacts uncoated; resorbs over time to reduce chronic pressure; can carry steroid or neurotrophin.	Granted (US).
Internal drug channel in the electrode	US 6,309,410 (2001)	Advanced Bionics Corp.	Internal lumen/channel for drug delivery into the cochlea.	Granted (US).
Ionically conductive (hydrogel) layer on contacts	US 8,761,904 (2014); US 2011/0178587 A1	Cochlear Ltd.	Ionically conductive elastomer/hydrogel layer disposed into channels/grooves on contacts to enable ion transfer while resisting tissue overgrowth on the metal surface.	Granted (US) + family.

Continued on next page

Focus area	Representative filing	Assignee / Applicant	Key idea	Notes / status
Biologically integrated electrode devices (general)	US 8,005,526 (2011)	Regents of the Univ. of Michigan	PEDOT/biopolymer constructs with cell-templated topography (and live-cell hydrogel bioelectrodes) promoting intimate biological integration of electrodes.	Granted (US).
Mechanically anchored hydrogel layers (general)	US 2018/0132790 A1	Verily Life Sciences LLC	Mechanically anchoring hydrogels to noble-metal electrodes (voids, apertures, textured surfaces) to prevent delamination.	Application (US); related grants exist (e.g., US 10,736,572; US 10,729,901).
Zwitterionic anti-fouling coatings on CIs	US 11,525,069 (2022); US 11,814,526 (2023); US 12,116,498 (2024); AU 2017/280359 B2; JP 7,005,535 B2; CN 109562202 B	Univ. of Iowa Research Foundation (Hansen / Guymon)	"Durable photopolymerizable cross-linked anti-fouling coatings": photo-grafted, photo-polymerized zwitterionic hydrogel thin films (pCBMA / pSBMA) covalently bonded to PDMS-based CI biomaterials to resist protein/cell fouling and reduce intracochlear fibrosis.	Granted (US, AU, JP, CN); family active.
